# FGFR2b is essential for salivary gland duct homeostasis and MAPK-dependent seromucous acinar cell differentiation

**DOI:** 10.21203/rs.3.rs-2557484/v1

**Published:** 2023-02-16

**Authors:** Marit H. Aure, Jennifer M. Symonds, Carlos U. Villapudua, Joshua T. Dodge, Sabine Werner, Wendy M. Knosp, Matthew P. Hoffman

**Affiliations:** 1 Matrix and Morphogenesis Section, National Institute of Dental and Craniofacial Research, National Institutes of Health, Bethesda, Maryland 20892, USA.; 2 Institute of Molecular Health Sciences, Department of Biology, Swiss Federal Institute of Technology (ETH) Zurich, Zurich, Switzerland.

**Keywords:** FGFR1b, FGFR2b, FGF7, salivary gland, exocrine, seromucous acinar cells, basal duct cells, development, differentiation

## Abstract

Exocrine secretory acinar cells in salivary glands (SG) are critical for oral health and loss of functional acinar cells is a major clinical challenge. Fibroblast growth factor receptors (FGFR) are essential for early development of multiple organs, including SG. However, the role of FGFR signaling in specific epithelial SG populations later in development and during acinar differentiation are unknown. Here, we predicted FGFR dependence in specific populations using scRNAseq data and conditional mouse models to delete FGFRs in vivo. We identifed essential roles for FGFRs in craniofacial and early SG development, as well as progenitor function during duct homeostasis. Importantly, we discovered that FGFR2b was critical for seromucous and serous acinar cell differentiation and secretory gene expression (*Bpifa2* and *Lpo*) via MAPK signaling, while FGFR1b was dispensable. We show that FGF7, expressed by myoepithelial cells (MEC), activated the FGFR2b-dependent seromucous transcriptional program. We propose a model where MEC-derived FGF7 drives seromucous acinar differentiaton, providing a rationale for targeting FGFR2b signaling in regenerative therapies to restore acinar function.

## Introduction

Exocrine glands secrete fluids essential for maintenance of their target tissues^1^. Exocrine SGs are critical for oral health as they generate saliva for mastication, maintenance of the oral microbiome, and lubrication of the oral cavity. There are three major mammalian SGs, submandibular (SMG), sublingual (SLG), parotid (PG) as well as minor salivary glands (MSG) which all differentiate in the composition of saliva they secrete^2,3^. The bulk of saliva secretion comes from highly specialized acinar cells defined by the secretory proteins they produce and are either seroumucous, mucous or serous depending on the protein contents of the saliva. Despite the central role of acinar cells, not much is known about the developmental mechanisms that drive acinar cells to either produce a mucous containing saliva compared with watery serous saliva. In vivo, acinar cells are surrounded by contractile myoepithelial cells (MEC) that wrap around and directly contact the acini, and connected duct cells that modify and transport saliva into the oral cavity. Loss of acinar cells is a common feature of pathologies including autoimmune diseases and as a side effect of irradiation therapy, and acinar regeneration continues to be a major clinical challenge. Preclinical studies have shown through lineage tracing that acinar and MECs are self-maintained, while basal duct cells are restricted progenitors during homeostasis^4^. Following injury, all cell compartments have regenerative potential, however, the contribution from any lineage is dependent on the degree and type of injury^4^. This has resulted in an updated view of stemness and plasticity in SGs leading to a renewed focus on niche signals and microenvironments that may inform regenerative therapies^5^.

Fibroblast growth factor receptors (FGFR) are a family of four receptor tyrosine kinases involved in development, progenitor cell proliferation, and tumorigenesis of multiple organ systems including SGs^6,7^. The critical role for FGFR signaling in human SGs is evident by haploinsufficiency of either FGFR2b or one of its major ligands, FGF10, which leads to two rare genetic diseases, aplasia of lacrimal and salivary glands (ALSG: MIM #180929) and lacrimo-auriculo-dento-digital syndrome (LADD: MIM #149730)^8^. Studies using murine models have established that both FGFR1b and FGFR2b are expressed in the epithelium during embryonic development and the ligands, FGF10 and FGF7, are expressed in the mesenchyme. Paracrine signaling between FGF10 in the mesenchyme and FGFR2b in the epithelium is required and sufficient for SG initiation, while FGF7 is dispensable for gland initiation^9–11^. Reduction in FGFR1b signaling leads to decreased branching morphogenesis and smaller glands^12^. Hypoplastic glands are found in *Fgf10*^+/−^ and *Fgfr2b*^+/−^ mice^13,14^, while ligand-dependent-gain-of-function, due to an *Fgfr2b* mutation (*Fgfr2*^+/Neo-S252W^) leads to hyperplasia^15,16^, suggesting sensitivity to FGF and FGFR protein expression levels. Although SGs, like many other branched organs, are dependent on FGFR signaling for early development, the role of FGFRs in cell-specific lineages, progenitor function and differentiation of specific acinar cell types are not known.

To investigate the role of FGFR signaling in specific SG cell types, we leveraged existing single-cell RNA-sequencing (scRNAseq) datasets^17–20^ and confirm that human and mouse SG have similar expression patterns of both FGFR and FGF ligands. We used mice carrying floxed FGFR alleles with epithelial Cre drivers to conditionally delete FGFRs in specific cell populations in vivo. We confirmed the essential role of FGFR2b in the primary endbud for gland initiation and identified requirement of FGFRs in adult duct progenitor function. We discovered that FGFR2b signaling drives seromucous and serous acinar differentiation in the SMG and SLG, respectively, while FGFR1b is dispensable. Further investigation using ex vivo organ culture and loss and gain of function approaches identified the FGFR signaling was via MAPK signaling and the seromucous acinar transcriptional program was stimulated by FGF7, which is produced by adult MECs.

## Results

### *Fgfr1b* and *Fgfr2b* are enriched in basal duct, acinar, and MECs during postnatal development in mouse SMG

**Figure 1:**
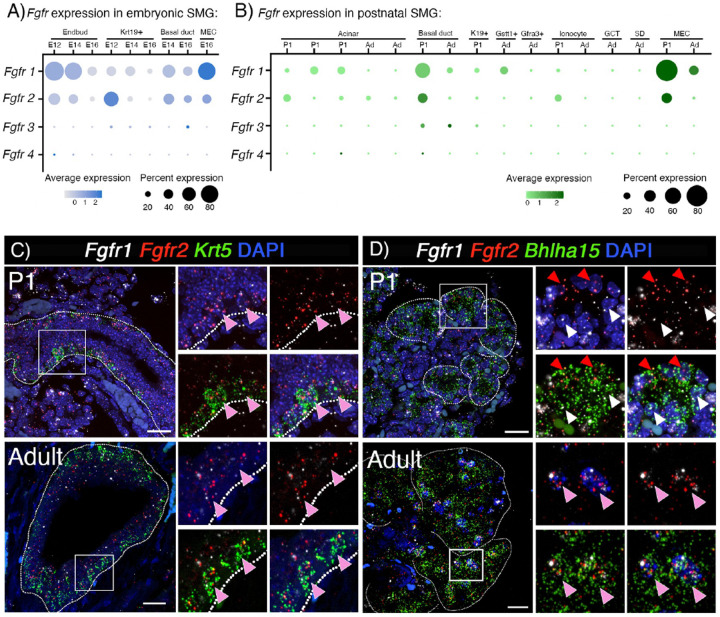
*Fgfr1b* and *Fgfr2b* are enriched in basal duct, acinar and MEC during postnatal development in mouse SMG. (A) Dotplot of scRNAseq data show that *Fgfr1b* and *Fgfr2b* are enriched in epithelial cell populations at E12. At E14 and E16, expression of both receptors decrease in Krt19+ duct and endbud, while it is maintained in basal duct and MECs. (B) *Fgfr1b* and *Fgfr2b* are enriched in acinar cells, basal duct, and MECs in postnatal glands. (C) In situ hybridization with *Fgfr1b* (white), *Fgfr2b* (red), and *Krt5* (green) show enrichment in basal duct cells in SMGs from P1 and adult (pink arrowhead). Scale bar: 20 μm. (D) In situ hybridization with *Fgfr1b* (white), *Fgfr2b* (red), and *Bhlha15* (green) show enrichment in acinar cells at both P1 and adult. P1 acinar cells were either *Fgfr1b* (red arrowhead) or *Fgfr2b* (white arrowhead), while adult acinar cells have both receptors (pink arrowhead). Scale bar: 20 μm.

We propose that understanding the functional roles of FGFRs in specific adult cells in the SG will provide important mechanistic insight for developing therapeutic strategies. Thus, we first utilized the recently generated scRNAseq atlas of murine salivary gland development to map the cell-specific expression of all FGFRs^17^. The scRNAseq atlas contains several stages of embryonic (E12, E14 and E16) and postnatal development. For the postnatal analysis, we combined data from postnatal day 30 (P30) and P300 and refer to these combined stages as adult. As expected in embryonic glands, both *Fgfr1b* and *Fgfr2b* were primarily expressed in the endbud (88% of cells in cluster) and Krt19+ duct (65% of cells in cluster), respectively (Figure 1A). *Fgfr3* was detected in 5–7% of duct cells and *Fgfr4* was barely detectable in E12 endbuds (~1%). Postnatally, *Fgfr1b* and *Fgfr2b* were both expressed in basal duct (*Fgfr1b*:16–55%, *Fgfr2b*: 32–4%), MECs (*Fgfr1b*:31–82%, *Fgfr2b*: 3–34%) and the acinar cell populations previously described (*Fgfr1b*: 1–26%, *Fgfr2b*: 5–23%) (Figure 1B). *Fgfr1b* was also detected in Gstt1+ intercalated duct (25%) (Figure 1B). *Fgfr3* was detected in ~9% of basal duct cells and *Fgfr4* was detected in <1% of basal ducts and acinar cells. This expression pattern was similar to that of adult human SMG, MSG, and PG (Figure S1A)^18–20^.

We focused our analysis on *Fgfr1b* and *Fgfr2b* due to their higher expression in both embryonic and postnatal development, compared to other *Fgfr* genes. In situ hybridization in postnatal glands confirmed the enrichment in *Krt5+* cells, which include basal ducts and MECs (Figure 1C, Figure S1B). There was also widespread *Fgfr1b* and *Fgfr2b* coexpression with *Bhlha15+* (MIST1), a canonical acinar cell marker (Figure 1D). Notably, at P1, acinar cells were enriched for either *Fgfr1b* or *Fgfr2b*, while adult acinar cells were positive for both receptors (Figure 1D).

Taken together, these data show that *Fgfr1b* and *Fgfr2b* are the most widely expressed FGFRs in SG epithelium. They are enriched in MECs, basal duct and acinar cells, all populations which have progenitor potential during regeneration and have been shown to self-renew during adult SG homeostasis. Based on these findings, we predicted that both FGFR1B and FGFR2B signaling are important for SG epithelial cell development and critical in specific lineages and cell populations at later stages of development. We sought to test this hypothesis using bioinformatic data to direct studies with genetic mouse models and explant culture.

### Genetic deletion of *Fgfr1b and Fgfr2b* in the ectoderm (Crect) and epithelium (Krt14Cre) highlight their distinct roles in SG organogenesis as well as in limb and craniofacial development in mice.

Based on previous global FGFR knockout studies showing differential roles for FGFR1b and FGFR2b at different stages of development, we predicted that deleting *Fgfr1b* in the entire E12 epithelium would not affect gland initiation, while *Fgfr2b* would be required.

**Figure 2:**
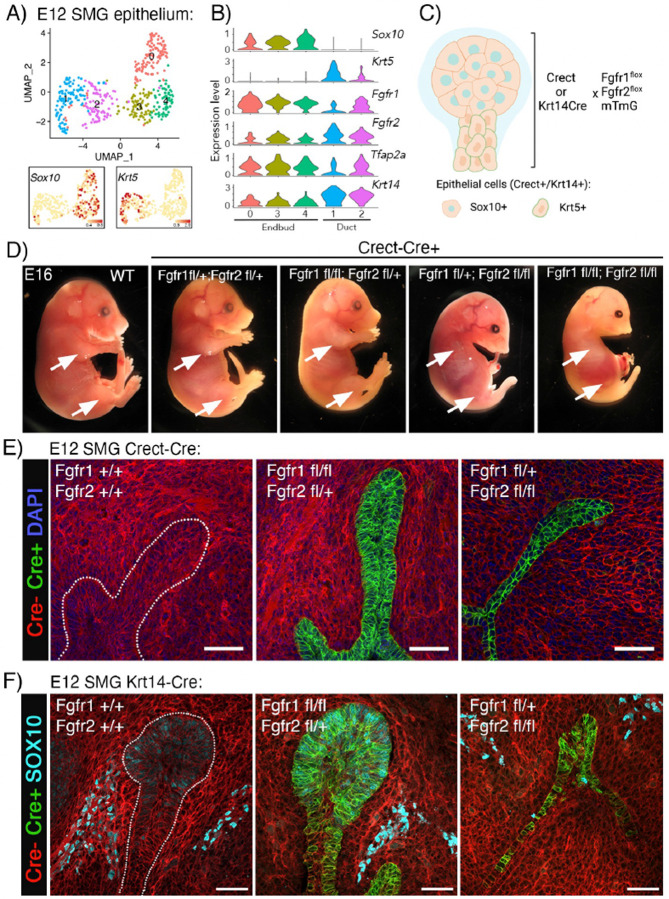
*Fgfr1b and Fgfr2b* are required for SMG and craniofacial development. (A) UMAP showing E12 SMG epithelium grouped into endbud (clusters 0, 3 and 4) and duct (clusters 1 and 2) based on *Sox10* and *Krt5* expression, respectively. (B) Violin plots showing enrichment of *Fgfr1b*, *Fgfr2b*, *Tcfap2a (Crect), and Krt14* in Sox10+ endbud and Krt5+ duct. (C) Strategy for *Fgfr1b* and *Fgfr2b* deletion in SG epithelium. Created using Biorender.com. (D) Gross images of *Crect; Fgfr1*^*fl*^*;Fgfr2*^*fl*^ embryos at E16. *Crect+;Fgfr1*^*fl*^+;*Fgfr2*^*fl/fl*^ embryos failed to develop limbs (arrows) and had cleft palate and maxillar and mandibular hypoplasia. This was exacerbated upon the loss of an additional *Fgfr1b* allele. Embryos that are heterozygous for *Fgfr2b* are grossly normal and indistinguishable from *Crect-* littermate controls. (E) Deletion of *Fgfr1b* and one copy of *Fgfr2b* was comparable to WT glands, while *Fgfr2b* deletion in Crect+ epithelial cells (green) impede SMG and SLG development (Cre- cells are red), scale bars: 50 μm. (F) Deletion of *Fgfr1b* in Krt14+ cells did not affect endbud formation, while *Fgfr2b* deletion led to loss of Sox10+ endbud cells. Cre+: green, Cre-: red, Sox10: Cyan. Scale bars: 50 μm.

Endbud and duct cells in the E12 epithelium can be identified bioinformatically by enrichment of *Sox10* and *Krt5*, respectively (Figure 2A, B). At this stage, both populations expressed *Fgfr1b* and *Fgfr2b* along with the ectodermal transcription factor AP-2 (*Tfap2a* or *Crect*) and the epithelial marker *Krt14* (Figure 2B). Based on these data, we used the ectoderm-specific *Tfap2a-Cre* (Crect) or the epithelial specific *Krt14Cre* mouse strains to delete *Fgfr1b* and *Fgfr2b* in vivo. Each of these strains were crossed with mice carrying floxed alleles for *Fgfr1b* and *Fgfr2b* in addition to the cell membrane-targeted, two-color fluorescent Cre-reporter mTmG mouse strain (Figure 2C). This generates embryos where Cre+ cells have *Fgfr* deletions and express cell membrane-localized GFP. Thus, for further analysis, GFP was used as a pseudomarker for *Fgfr* manipulation.

Both Cre models generated embryos with gross morphological changes that were non-viable due to lack of organ development upon *Fgfr2b* deletion. While *Crect+;Fgfr1*^*fl/+*^*;Fgfr2*^*fl/+*^ embryos were indistinguishable from *Crect-* littermate controls, *Crect+;Fgfr1*^*fl/+*^*;Fgfr2*^*fl/fl*^ embryos strongly phenocopied other *Fgfr2b* knockout transgenic mice. The embryos failed to develop limbs, had fused caudal vertebrae, cleft palate and maxillary and mandibular hypoplasia (Figure 2D, S2A). Interestingly, this phenotype was exacerbated upon the loss of an additional *Fgfr1b* allele suggesting additional roles for *Fgfr1b* in ectodermal tissues, especially for mandibular and craniofacial development (Figure 2D). Even though the embryos had major developmental issues, the mean weight was not significantly changed (Figure S1B). Deleting FGFRs in the Krt14+ lineage resulted in a milder phenotype; limbs developed in *Krt14Cre+;Fgfr1*^*fl/+*^*;Fgfr2*^*fl/fl*^, but defects in digitization of the hind limbs and eyelid formation were observed in E16 embryos (Figure S2C). Interestingly, there were no obvious craniofacial developmental defects with loss of *Fgfr2b* in the Krt14+ lineage. The *Krt14Cre+;Fgfr1*^*fl/fl*^*;Fgfr2*^*fl/+*^ also appeared similar to *Krt14Cre-* controls up to E16 (Figure S2C).

SGs from the E12 *Crect+;Fgfr1*^*fl/fl*^*;Fgfr2*^*fl/+*^ were comparable to control and formed a SMG with a stratified, invaginating epithelium in the condensing mesenchyme (Figure 2E). In embryos with loss of two alleles of *Fgfr2b*, initiation of both the SMG and SLG occurred, appearing as an infolding of the epithelium, but the primary endbud failed to enlarge. The fold in the oral epithelium appeared similar to an early E11 SMG, but failed to form a stratified endbud (Figure 2E). Similarly, *Krt14Cre+;Fgfr1*^*fl/fl*^*;Fgfr2*^*fl/+*^ glands were comparable to control while *Krt14Cre+;Fgfr1*^*fl/+*^*;Fgfr2*^*fl/fl*^ glands had a thickening of the oral epithelium, but had not stratified to form an enlarged endbud further shown by an almost complete absence of Sox10+ endbud cells (Figure 2F). Mesenchymal SOX10+ expression in neural crest cells, precursors to the parasympathetic ganglion, was not affected (Figure 2F). For both *Crect* and *Krt14-Cre* strains, no salivary gland was observed at E16, indicating that there was not simply a delay in development, but an absence of SG formation (data not shown).

Taken together, these data confirm the central role of *Fgfr2b* in gland initiation and demonstrate that *Fgfr1b* is dispensable for endbud formation. Further, to investigate cell specific roles in lineage specification, FGFRs must be deleted in duct and acinar cells directly using additional cre models.

### FGFR1b and FGFR2b signaling is required for postnatal duct progenitor lineage contribution and duct homeostasis

Next, we aimed to determine whether the expression of FGFRs in basal ducts is required for their development. Previous lineage tracing has showed *Sox10* expressing cells in the endbud at E12 give rise to most of the gland parenchyma^21^. Thus, we predicted that loss of *Fgfr1b* and *Fgfr2b* in the Krt5+ ducts (Sox10-negative) at E12 might be compensated for by Sox10+ lineage.

**Figure 3:**
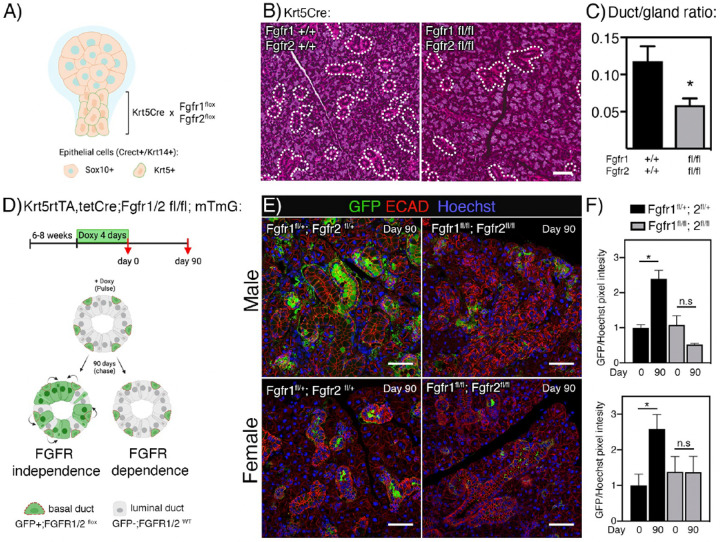
*Fgfr1b* and *Fgfr2b* are required for postnatal duct development and lineage contribution. (A) *Krt5*Cre mice were crossed with *Fgfr1*^*fl*^*;Fgfr2*^*fl*^ strains to ablate FGFRs in *Krt5* lineage during development. Created using Biorender.com. (B) Representative images of H&E staining from WT and *Krt5*Cre+;*Fgfr1*^*fl/fl*^*;Fgfr2*^*fl/fl*^ SMGs. Dotted lines indicate duct area. Scale bars: 50 μm. (C) Quantification of duct/gland ratio showed ductal hypoplasia after *Fgfr1b* and *Fgfr2b* deletion in Krt5+ lineage in adult SMGs. Unpaired t-test was used to calculate significance (*p<0.05). (D) Adult (6–8 weeks old) *Krt5rtTA;tetCre;Fgfr1/2*^*fl*^*; mTmG* mice were fed doxycycline for 4 days (pulse, day 0) and glands were analyzed after a 90-day chase. Increased GFP expression after FGFR deletion would indicate FGFR independence of progenitor function, while no change would indicate the opposite. Created using Biorender.com. (E) SMGs sections were stained for E-cadherin (ECAD, red) and GFP (green). Day 90 showed less GFP expression in samples where *Fgfr1b* and *Fgfr2b* were deleted compared to control. Scale bars: 50 μm. (F) Quantification of GFP expression in IHC sections at day 0 and day 90 showed reduced contribution from Krt5+ duct cells after *Fgfr1b* and *Fgfr2b* deletion in both male and female glands. Unpaired t-test was used to calculate significance between day 0 and day 90 for each genotype (*p<0.05).

Bioinformatic analysis of duct cells from E14, E16 and P1(Figure S2D) showed a clear enrichment of *Fgfr1b* and *Fgfr2b* in basal duct cells, while luminal duct cells were enriched for *Krt19*, *Cldn3* and *Cldn4* and Ionocytes were enriched for *Foxi1* (Figure S2D). Thus, *Fgfr1b* and *Fgfr2b* were specifically deleted in basal ducts by crossing the *Fgfr1b;Fgfr2* floxed mice with a constitutive *Krt5-Cre* mouse strain (Figure 3A). In this model FGFR1B and FGFR2B are deleted in E12 duct and Krt5+ lineage, while being expressed elsewhere in the gland (Figure 3A, S2D). Previous work has shown that this cross leads to viable mice; however, they develop a progressive skin phenotype^22^. Indeed, SGs were present in adult mice although they were 50% smaller in *Krt5cre+;Fgfr1*^*fl/fl*^*;Fgfr2*^*fl/fl*^ mice compared to wildtype (WT, Krt5Cre-) mice (Figure S2E). Upon histological examination, the *Krt5Cre+;Fgfr*^*fl/fl*^*;Fgfr2*^*fl/fl*^ SGs had distinct ductal hypoplasia with loss of granular convoluted tubules (GCT) and 60% reduction in the ratio of duct/total gland area (Figure 3B, 3C). GCTs are specialized ducts that are more abundant in male mice salivary glands and produce abundant NGF and EGF and these results points to a specific role for FGFR signaling in basal progenitors leading to GCT duct development or postnatal GCT differentiation.

To further dissect the role of FGFRs in duct lineages at later stages of development, we used the inducible Krt5rtTA;tetCre mice crossed with *Fgfr1*^*fl/fl*^*;Fgfr2*^*fl/fl*^ and mTmG mice. These mice carry a tetracycline transactivator gene that induces Cre expression in Krt5+ basal cells. We analyzed the effect of FGFR deletion in the Krt5 lineage during embryonic SG duct development by feeding doxycycline to pregnant females and harvested glands from pups at P1 (Figure S2F). Additionally, we studied postnatal duct lineage by performing lineage tracing experiments in adult mice.

Deletion of *Fgfr1b* and *Fgfr2b* from the Krt5-cell lineage during embryonic development resulted in no significant change in P1 gland weight normalized to body weight (Figure S2F). Gross histology was also normal in Krt5rtTA;tetCre+; *Fgfr1*^*fl/fl*^*;Fgfr2*^*fl/fl*^ glands compared to WT (Cre-) (Figure S2G). Gene expression analysis showed a reduced trend in expression of ductal markers although this trend was not significant (Figure S2H). This suggests that FGFR signaling in Krt5+ basal cells prior to birth are either not involved in lineage contribution and differentiation of duct populations, or that loss of *Fgfr1b* and *Fgfr2b* signaling in the ducts (Krt5+ cells) can be compensated for by other pathways. It also suggests that the duct phenotype observed in the non-inducible model was likely due to postnatal differentiation events.

To address the potential role of FGFR1B and FGFR2B in basal duct lineage contribution to GCTs, adult *Krt5rtTA;tetCre+; Fgfr1*^*fl/fl*^*;Fgfr2*^*fl/fl*^*; mTmG* mice (male and female, 6–8 weeks) were fed doxycycline for 4 days (pulse, day 0) resulting in FGFR deletion and onset of GFP reporter expression (Figure 3D). After FGFR deletion, we predicted that lineage tracing similar to control would indicate FGFR independence of progenitor function, while decreased amount of lineage tracing would indicate the opposite (Figure 3D). After a 90-day chase, lineage tracing from Krt5+ duct cells was clearly evident in control glands (*Krt5rtTA;tetCre+;Fgfr1*^*fl/+*^*;Fgfr2*^*fl/+*^*;mTmG*), while this pattern was not seen in glands from *Krt5rtTA;tetCre+;Fgfr1*^*fl/fl*^*;Fgfr2*^*fl/fl*^*;mTmG* mice (Figure 3E). Quantification of GFP positive cells showed similar baseline GFP levels in both control and *Fgfr1b;Fgfr2b* deletion, although expression in males were higher than females (comparison not shown) (Figure 3F). While quantification of GFP expression showed significant increase after 90 days chase, no significant increase of GFP was seen after *Fgfr1b;Fgfr2b* deletion in basal duct cells (Figure 3F). These results show that FGFR1B and FGFR2B signaling is required for basal duct contribution to duct homeostasis in postnatal glands in both males and female SGs.

### *Fgfr1b* and *Fgfr2b* are differentially enriched in specific acinar subpopulations during gland development

Next, we set out to investigate whether FGFRs are required for specific types of acinar differentiation because our initial analysis showed *Fgfr1b* and *Fgfr2b* expression in both embryonic endbuds and postnatal acinar cells (Figure 1A, D). Acinar specification starts around E15 with onset expression of the canonical acinar markers *Aqp5*, *Bhlha1*5 and *Cldn10*^17,23,24^. Acinar cells are defined as serous, seromucous or mucous based on their secretory products and we previously reported that at birth, two subpopulations of acinar cells are identified by *Smgc* and *Bpifa2* expression^17^. In P1 SMG, *Bpifa2+* acinar cells are transcriptionally overlapping with mature seromucous acinar cells, while the *Smgc+* population is overlapping with adult *Gstt1+* intercalated ducts^17^.

**Figure 4:**
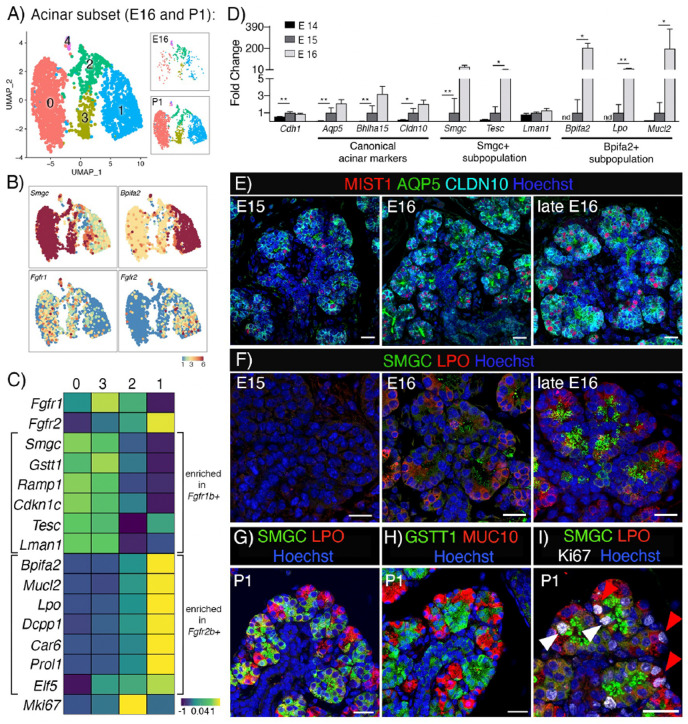
*Fgfr1b* and *Fgfr2b* are differentially enriched in specific acinar subpopulations during gland development. (A) UMAP showing E16 and P1 acinar cells. This dataset was used to analyze *Fgfr* expression and transcriptional profiles of the acinar subpopulations. (B) UMAPs showing expression of *Smgc*, *Bpifa2*, *Fgfr1b*, and *Fgfr2b* in E16 and P1 acinar cells. (C) Heatmap showing average expression of genes enriched in *Fgfr1b*+ and *Fgfr2b*+ acinar populations. (D) Onset of acinar markers and subpopulations in vivo occurs around E15 (n=3, normalized to E15 and housekeeping gene *Rsp29*, nd = not detected. One-way ANOVA with Dunnett’s test for multiple comparisons (**p<0.0099, *p<0.04). (E) Acinar markers AQP5, MIST1 and CLDN10 can be detected from E15. Scale bars: 50 μm. (F) Protein expression of SMGC and LPO can be detected by E16. Scale bars: 20 μm. (G-H) The two populations can be visualized by SMGC and LPO or GSTT1 and MUC10 in P1 SMG. Scale bars: 20 μm. (I) Proliferating Ki67+ cells (white) detected in both SMGC (green) and LPO (red) cells in P1 SMG. Scale bar: 20 μm.

To further analyze FGFR expression during acinar differentiation we bioinformatically isolated and re-clustered acinar cells from E16 and P1, resulting in 4 distinct clusters (Figure 4A and Figure S3A). Cluster 4 expressed relatively low levels of the canonical acinar markers and had additional genes from multiple cell lineages that do not align with acinar cell identity including, *Krt14*, *Krt5*, *Trp63*, *Acta2*, *Col1a1* and *Col3a1*. Although doublets were previously removed from the dataset, it is not clear whether this small cluster are remaining doublets or cells in a transitional cell state. Due to this ambiguity, this cluster was excluded from further analysis.

Clusters 0 and 3 correspond to *Smgc+* acinar cells and were enriched for *Fgfr1b*, while cluster 1 corresponded to *Bpifa2+* acinar cells and were enriched for *Fgfr2b* (Figure 4B). *Fgfr1b+/Smgc+* cells were also enriched for *Gstt1, Ramp1, Cdkn1c, Tesc* and *Lman1* while *Fgfr2b+/Bpifa2+* cells were enriched for *Mucl2, Lpo, Dcpp1, Car6, Prol1*and *Elf5* (Figure 4C). In general, all markers had higher expression level at P1 compared to E16. *Smgc*, *Bpifa2* and *Lpo* were enriched in their respective populations at both E16 and P1; however, some markers were stage-specific, such as *Tesc* at E16 and *Gstt1* at P1 in *Fgfr1b*+ cells and *Mucl2*, *Car6* and *Prol1* in *Fgfr2b*+ cells at P1 (Figure S3B).

The onset of acinar specification and the relative expression of markers for the specific populations were confirmed at three timepoints (E14, E15 and E16) using qPCR (Figure 4D). Immunostaining for canonical acinar specification markers showed detection at E15 with a progressively increasing organization of luminal AQP5, basolateral CLAUDIN10 (CLDN10) and nuclear MIST1 (Figure 4E). Markers for the two subpopulations could be detected from E16, and by late E16 both populations were clearly distinguished with immunostaining for SMGC and LPO proteins (Figure 4F). Furthermore, in P1 glands, the two pro-acinar populations could be visualized through immunostaining with either SMGC and LPO or GSTT1 and MUC10 (Figure 4G, H). Co-staining of cells with SMGC and GSTT1 or co-staining with LPO and MUC10 further confirmed they were expressed in the same cell populations (Figure S3C, D). Based on average expression, cluster 2 (Figure 4C) appeared to be double positive for *Fgfr1b* and *Fgfr2b*; however, this cluster contained cells either *Smgc+* or *Bpifa2+*, and was defined by proliferative markers such as *Mki67*, *Bub1b*, *Aurka* and *Top2a* (Figure S3E). Pathway analysis indicated active proliferation as the major functional state of these cells (Figure S3E). Furthermore, proliferating acinar cells were found within both subpopulations (clusters 0 and 1, Figure 4I), indicating that cluster 2 is mainly proliferating cells made up by a mix of the two distinct populations rather than a *Fgfr1b* and *Fgfr2b* double positive population (Figure 4I). Taken together, *Fgfr1b* and *Fgfr2b* are enriched within specific acinar subpopulations, and we hypothesized that FGFR signaling would differentially affect expression of defining markers and either the development or maturation of these acinar subpopulations.

### *Fgfr2b* is required for seromucous and serous acinar differentiation through MAPK pathway

To test the direct effect of FGFR signaling on acinar differentiation, we used the Aqp5-Cre-IRES-dsRed mouse strain (ACID-Cre) in combination with *Fgfr1*^*fl/fl*^*;Fgfr2*^*fl/fl*^*;tdTomato* mice. Since AQP5 is expressed in several cell types including type I pneumocytes of the lung^25^, *Fgfr2b* deletion led to a lethal lung phenotype and *ACID-Cre+;Fgfr2*^*fl/fl*^ animals did not survive more than three days after birth (data not shown). Due to the postnatal lethal phenotype, we analyzed acinar development at P1-P3 in this model.

**Figure 5:**
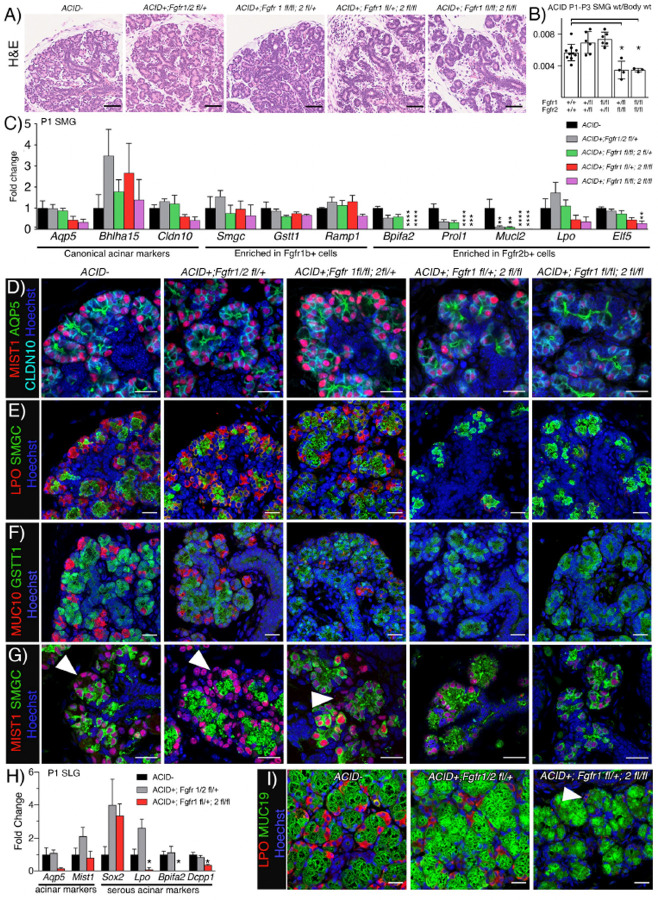
SMG seromucous and SLG serous acinar cell differentiation is FGFR2b-dependent. (A) Deletion of *Fgfr2b* led to acinar atrophy in P1 SMGs compared to either WT or *Fgfr1b* deletion. Scale bars: 50 μm. (B) *Fgfr2b* deletion in acinar cells leads to a decrease in gland weight ratio compared to wild type (Cre-). Graph shows mean ratio of gland weight over body weight with SD, n ≥3. One-way ANOVA with Dunnett’s test for multiple comparisons to control (*p<0.05). (C) Canonical acinar genes and genes enriched in *Fgfr1b*+ population were not affected by deletion of either *Fgfr1b*, Fgfr2b or both. Genes enriched in *Fgfr2b*+ cells were decreased after deletion of *Fgfr2b* or both receptors (n ≥3 for each genotype). One-way ANOVA with Dunnett’s test for multiple comparisons to WT control (****p<0.001, ***p =0.0003,**p<0.003). (D) AQP5 (green), MIST1 (red), and CLDN10 (cyan) was detected in P1 SMGs from all groups. Scale bars: 20μm. (E) Ablation of either Fgfr1b, Fgfr2b, or both did not affect localization pattern of SMGC (green), while LPO (red) was not detected after *Fgfr2b* deletion in P1 SMGs. Scale bars: 20μm. (F) Deletion of either *Fgfr1b*, *Fgfr2b*, or both did not affect localization pattern of GSTT1 (green), while MUC10 (red) was not detected after *Fgfr2b* deletion in P1 SMGs. Scale bars: 20μm. (G) MIST1 (red) and SMGC (green) staining showed overlap in all MIST1 cells after *Fgfr2b* deletion. Scale bar: 20μm. Arrowheads indicating MIST1+/SMGC- cells. (H) qPCR showing loss of serous acinar markers in SLGs after *Fgfr2b* ablation. One-way ANOVA with Dunnett’s test for multiple comparisons to control (*p<0.05). (I) IHC showed decreased staining of LPO (red) and sustained MUC19 (green) after *Fgfr2b* deletion. Arrowhead pointing to low LPO expressing cell. Scale bars: 20 μm.

In ACID-Cre+ glands, gross histology of *Fgfr1*^*fl/+*^*;Fgfr2*^*fl/+*^ and *Fgfr1*^*fl/fl*^*;Fgfr2*^*fl/+*^ SMGs were comparable to control, while both *Fgfr1*^*fl/+*^*;Fgfr2*^*fl/fl*^ and *Fgfr1*^*fl/fl*^*;Fgfr2*^*fl/fl*^ had pronounced acinar hypoplasia (Figure 5A). This was reflected in gland size, where both *Fgfr1*^*fl/+*^*;Fgfr2*^*fl/fl*^ and *Fgfr1*^*fl/fl*^*;Fgfr2*^*fl/fl*^ were ~40% smaller compared to WT (Figure 5B). Despite the severe acinar hypoplasia, gene expression of the canonical markers *Aqp5*, *Bhlha15 and Cldn10* were only moderately decreased (Figure 5C). Accordingly, AQP5, MIST1 and CLDN10 protein were detected by immunostaining in all genotypes, although the organization of the acini was severely affected by *Fgfr2b* deletion (Figure 5D). Expression levels of defining genes for both acinar subpopulations showed no change in *Smgc, Gstt1*, or *Ramp1* (all defining genes for *Fgfr1b*+ cells), while there was reduced expression of *Bpifa2*, *Prol1*, *Mucl2, Lpo*, and *Elf5* after *Fgfr2b* deletion (Figure 5C). Interestingly, *Bpifa2*, *Prol1, and Mucl2* were also decreased in *Fgfr1*^*fl/+*^*;Fgfr2*^*fl/+*^ and *Fgfr1*^*fl/fl*^*;Fgfr2*^*fl/+*^. This decrease was due to heterozygous *Fgfr2b* rather than deletion of *Fgfr1b* as *Fgfr1*^+/+^*;Fgfr2*^*fl/+*^ SMGs, gave similar results (data not shown). In line with the gene expression, protein detection of the two populations using either SMGC and LPO or GSTT1 and MUC10 showed loss of LPO and MUC10 after loss of *Fgfr2b* expression (Figure 5E, 5F). Further, all acinar cells (MIST1+) expressed SMGC after FGFR2B deletion, indicating a complete loss of seromucous acinar differentiation (Figure 5G).

SLG acini consist of proximal mucous cells expressing MUC19 and distal serous cells expressing LPO. FGFR2b signaling was important in the SLG as evidenced by the loss of serous cells after *Fgfr2b* deletion (Figure S4A-C). *Fgfr2b* deletion did not change the canonical acinar markers, *Aqp5* and *Bhlha15*, while serous markers *Lpo*, *Bpifa2* and *Dcpp1* were reduced (Figure 5H). Interestingly, *Sox2*, a potency marker in SLG acinar cells was increased in expression, suggesting there may be increased Sox2+ progenitors when serous acinar differentiation is reduced due to lack of FGFR2B-dependent differentiation (Figure 5H). Immunostaining supported this finding and there was a clear decrease of LPO staining in *Fgfr1*^*fl/+*^;*Fgfr2*^*fl/fl*^ glands while MUC19 staining in mucous cells was evident in all genotypes (Figure 5I). Taken together, this data shows that *Fgfr1b* is dispensable while *Fgfr2b* is required for differentiation and expression of secretory markers in seromucous and serous cells in the SMG and SLG, respectively. It also highlights that mucous acinar cell differentiation is independent of FGFR signaling.

**Figure 6:**
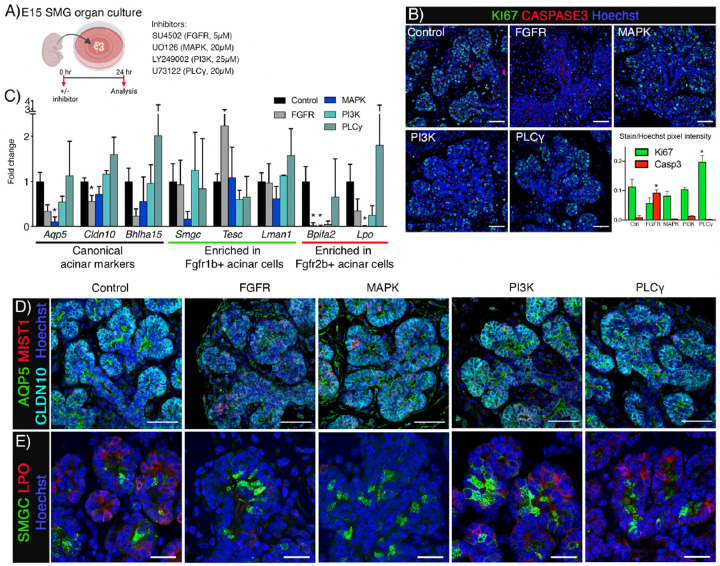
*Fgfr2b* is required for seromucous acinar differentiation through MAPK pathway. (A) E15 SMGs were treated with inhibitors and cultured ex vivo for 24 hrs before analysis. Created using Biorender.com. (B) Representative images showing Ki67 and Cleaved Caspase 3 in E15 SMG + 24 hrs. Scale bar: 20 μm. Quantification of Ki67 and Cleaved Caspase 3 staining showed significant increase in apoptosis after FGFR inhibitor. Treatment with PLCγ inhibitor increased proliferation, while MAPK or PI3K inhibitors showed no change. One-way ANOVA with Dunnett’s test for multiple comparisons to control (*p<0.05). (C) Gene expression of canonical acinar markers were decreased after FGFR inhibition. *Cldn10* and *Bpifa2* were decreased after FGFR inhibitor. *Aqp5, Bpifa2* and *Lpo* were also decreased after MAPK inhibitor. Genes enriched in the *Fgfr1b*+ population did not significantly change with the various inhibitors compared to vehicle control. One-way ANOVA with Dunnett’s test for multiple comparisons to control (*p<0.05). (D) Acinar differentiation was evident in all groups shown through IHC of AQP5, MIST1 and CLDN10. Scale bars: 50 μm. (E) After 24hrs, SMGC could be detected in all groups, while LPO was not detected after FGFR and MAPK inhibitor treatment. Scale bars: 20 μm.

We utilized WT mice (ICR) and an organ culture system to further manipulate downstream signaling required for FGFR-dependent seromucous acinar differentiation. Isolated E15 SMGs from ICR mice were cultured for 24 and 48 hrs to establish the baseline gene expression during culture conditions (Figure S5A). This showed consistent expression of *Fgfr1b, Fgfr2b, Fgf10* and *Fgf7*, and the downstream effectors *Etv4*, and *Etv5* (Figure S5B). We could also detect continued acinar differentiation ex vivo, reiterating in vivo gene expression profiles of the canonical markers *Aqp5*, *Bhlha15* and *Cldn10* as well as specific marker for the two subpopulations including *Smgc*, *Tesc*, *Lman1*, *Bpifa2*, *Lpo* and *Mucl2* (Figure S5C). Acinar cells were evident by staining of AQP5, MIST1 and CLDN10 and the two populations could be visualized by SMGC and LPO staining after 24hrs in culture, comparable to in vivo localization pattern (Figure S5D and S5E). For loss-of-function experiments using chemical signaling inhibitors we isolated E15 SMGs and cultured them with inhibitors for 24 hrs (Figure 6A). Based on the concentrations of inhibitors used in SMG organ culture experiments in previously reported studies, we treated glands with inhibitors for FGFR and its canonical downstream pathways. Specifically, we treated glands with a pan-FGFR inhibitor (SU5402, 5 μM), a MAPK inhibitor (UO126, 20 μM), a PI3K inhibitor (Ly249002, 25 μM) a PLCγ inhibitor (U73122, 25μM) or vehicle control (DMSO). We initially analyzed apoptosis (Cleaved-caspase3) and proliferation (Ki67) by immunostaining of SMG 24 hours after inhibitor treatment. Pan-FGFR inhibitor treatment upregulated apoptosis with a concomitant reduction in proliferation compared to vehicle control (Figure 6B). Treatments inhibiting either MAPK or PI3K did not affect proliferation or apoptosis, while PLCγ significantly increased proliferation (Figure 6B). These data show that changes in gene expression are not directly due to changes in cell survival and proliferation after MAPK or PI3K inhibitor treatment.

Expression of *Fgfr1b* and *Fgfr2b*, the ligands *Fgf10* and *Fgf7* or the epithelial marker *Cdh1* was not affected by any of the inhibitors (Figure S5 F). However, downstream effectors *Etv4* and *Etv5* were reduced after MAPK, PI3K or PLCg inhibitor treatment confirming inhibition of the pathway (Figure S5 F). *Bhlha15*, *Cldn10*, and *Aqp5* expression was reduced after FGFR inhibition. *Aqp5* expression was also reduced after MAPK inhibition, indicating a specific MAPK signaling dependence for *Aqp5* (Figure 6C). As expected, *Smgc*, *Tesc* and *Lman1* expression were not negatively affected by inhibitor treatments (Figure 6C). *Bpifa2* was decreased after FGFR, MAPK and PI3K inhibitor treatment, while *Lpo* showed a similar trend, while PLCγ inhibitor treatment did not affect *Fgfr2b*-dependent genes (Figure 6C). Gross histology of all groups was comparable to control (Figure S5G). After 24hrs treatment, luminal AQP5, nuclear MIST1 and lateral CLDN10, were detected in all groups indicating that early acinar differentiation was not lost (Figure 6D). Staining for SMGC showed expression of the protein in all groups, while LPO was not detected after FGFR or MAPK inhibitor treatment (Figure 6E). These results show that differentiation of seromucous acinar cells requires FGFR2b signaling through the MAPK pathway.

### Seromucous acinar transcriptional program can be activated by FGF7, which is expressed by MEC that contact acini, as well as FGF10 produced by fibroblasts

**Figure 7:**
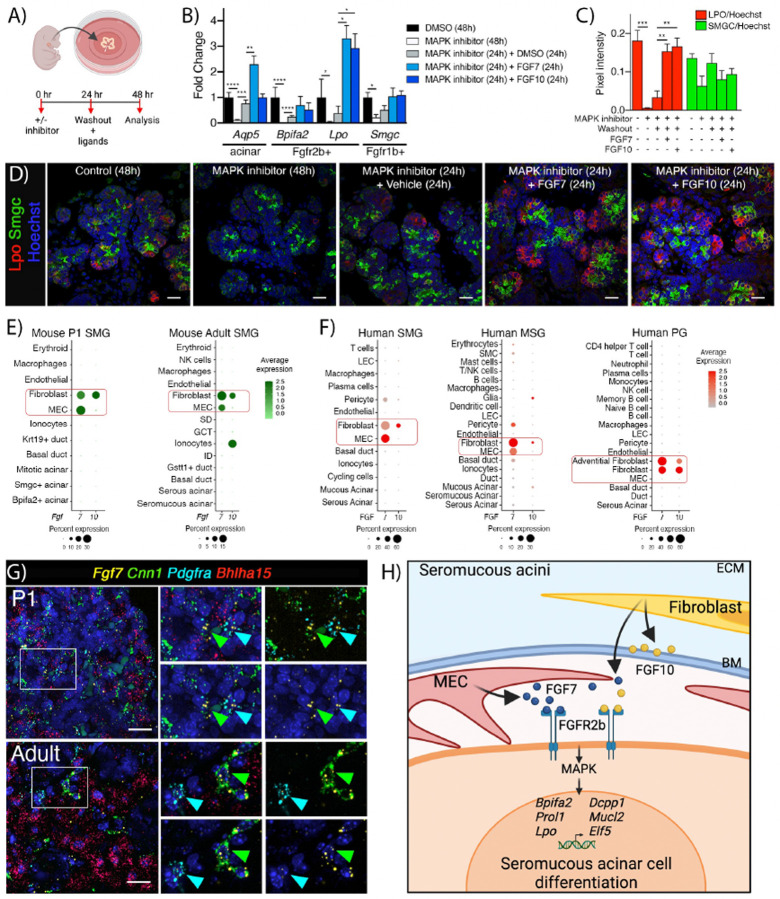
Acinar and MEC crosstalk via FGF7-FGFR2b signaling can activate seromucous transcriptional program. (A) Washout experimental timeline. Created using Biorender.com. (B) Genes reduced following MAPK inhibitor could be partially rescued by inhibitor washout. *Aqp5* and *Lpo* were further increased by FGF7 and FGF10 treatment (n=4). One-way Anova with Dunnett’s test for multiple comparisons (***p<0.0001, *p<0.03). (C) Quantification of IHC of LPO (green) and SMGC (red) in glands in washout experiment showed rescue of LPO by addition of FGF7 and FGF10 (n = 3–4). One-way Anova with Tukey test for multiple comparisons (***p<0.0003, **p<0.004). (D) Representative images of LPO (red) and SMGC (green) in E15 + 48 hrs organ culture with inhibitor, washout and ligand treatment as indicated. Scale bar= 20 μm. (E) DotPlots showing expression of *Fgf7* and *Fgf10* in postnatal mouse SMG from scRNAseq data. (F) DotBlots showing FGF7 and FGF10 in human SMG, MSG and PG from scRNAseq. (G) In situ hybridization with *Fgf7* (yellow), *Cnn1* (MECs, green), *Pdgfra* (fibroblasts, cyan), and *Bhlha15* (acinar cells, red), show enrichment of *Fgf7* in MECs and some in fibroblasts in both P1 and adult mouse SMG. Scale bar = 20 μm. (H) Crosstalk between acinar cells and MEC-derived FGF7 as well fibroblasts via FGF7/FGF10-FGFR2b drives seromucous acinar cell differentiation. MEC: myoepithelial cell, ECM: Extracellular matrix, BM: Basement membrane. Created using Biorender.com.

Both FGF7 and FGF10 are major ligands for FGFR2b signaling^26,27^ and activation of the acinar transcriptional program has therapeutic potential for regenerating exocrine secretory cells following injury or disease. Accordingly, we asked whether FGF7 and FGF10 could increase expression of *Fgfr2b*-dependent secretory markers. Initial experiments using E15 organ culture treated with additional exogenous FGF7 or FGF10 showed no increases in acinar gene expression compared to vehicle after 6 hrs, likely due to the robust endogenous FGF production (data not shown). Therefore, we performed a gain-of-function experiment to test whether the reduced seromucous differentiation after MAPK inhibition could be restored or increased by addition of exogenous FGF7 or FGF10. E15 glands were treated with MAPK inhibitor for 24 hrs before changing to fresh media with either MAPK inhibitor, the ligands FGF7 or FGF10 or a DMSO vehicle control (Figure 7A). Treatment with MAPK inhibitor for 48 hrs decreased *Aqp5*, *Bpifa2* and *Lpo* and washout with media containing vehicle control increased and partially rescued expression of all three genes (Figure 7B). However, addition of FGF7 further increased the expression of both *Aqp5* and *Lpo* above control expression, while *Bpifa2* expression was restored to control levels (Figure 7B). Addition of FGF10 did not increase *Aqp5* but did increase *Lpo* expression (Figure 7B). Inhibitor treatment for 48 hrs decreased expression of *Smgc* and addition of ligands showed an increasing trend although not significant compared to washout alone (Figure 7B). Furthermore, immunostaining confirmed an increase of LPO staining following stimulation with either FGF7 or FGF10 after MAPK inhibition. In contrast, SMGC immunostaining decreased, and washout reversed this although ligand treatment did not result in further increases compared with washout alone (Figure 7C, D). These results show that MAPK-dependent seromucous differentiation (LPO expression and protein staining) can be stimulated ex vivo by both FGF7 and FGF10.

Since activation of FGFR2b is critical for seromucous differentiation, we asked which cells are the in vivo source of these ligands. The acinar niche, or microenvironment contains multiple cells producing the signals needed for homeostatic acinar function. Signals from MECs, nerves, blood vessels, immune cells, and fibroblasts all contribute to the acinar niche. Both *Fgf7* and *Fgf10* were detected by scRNAseq in cells within the acinar niche. As expected, fibroblasts expressed both *Fgf7* and *Fgf10*, however surprisingly, *Fgf7* was also predominantly expressed in MECs, while *Fgf10* is expressed in mature ductal ionocytes and fibroblasts (Figure 7E), as recently reported^28^. In vivo, MECs directly contact and wrap around the acini, and this acinar complex is surrounded by the basement membrane, whereas, the stromal fibroblasts that produce FGF10 are separated from acini by the basement membrane. Interestingly, human SGs showed a similar expression pattern, with FGF10 in fibroblasts and FGF7 expressed in MECs of SMG and MSG, but not detected by scRNAseq in PG MECs (Figure 7F). We confirmed the novel expression of *Fgf7* in MECs and fibroblasts in both P1 and adult SMGs through in situ hybridization (Figure 7G). This suggests that FGF7 from MECs, in direct contact to acini, may provide critical niche signals for seromucous acinar differentiation. Taken together, we have shown that differentiation of seromucous acinar cells is dependent on FGFR2b-MAPK signaling via FGF7 and FGF10 from MECs as well as fibroblasts (Figure 7H).

## Discussion

We have previously defined roles for FGFR signaling during fetal ex vivo SMG branching morphogenesis in organ explant culture^29^. Here, we used genetic tools to conditionally delete FGFRs in vivo, identifying essential and cell-specific roles for FGFR signaling during gland initiation, duct homeostasis and seromucous and serous acinar differentiation. Leveraging scRNAseq data to map FGFRs to specific cell types at both early stages of SG development and in postnatal cells, allowed for precise predictions of lineage behavior which were confirmed by the different outcomes of either deleting FGFRs in the entire ectodoerm (Crect), basal epithelium (Krt14Cre), ductal lineage (Krt5cre) and acinar lineage (ACID). As predicted, deleting FGFR2b in ectodermal and epithelial lineage lead to salivary agenesis, confirming the requirement of FGFRs for early SG development. Surprisingly, loss of epithelial FGFR1b in the absence of FGFR2b increased the severity of mandibular and maxillar hypoplasia in Crect+;*Fgfr1*^*fl/fl*^*;Fgfr2*^*fl/fl*^ embryos, suggesting a specific and additional functional role for FGFR1b in craniofacial development in the absence of FGFR2b. We identifed essential FGFR functions in progenitor lineage contribution and maturation of ducts. Importantly, we discovered that FGFR2b-MAPK signaling was critical for seromucous and serous acinar cell differentiation, in the SMG and SLG respectively, while FGFR1b was dispensable. We propose a model, where FGF7, expressed by MEC in contact with acinar cells, acitivates the FGFR2b-dependent seromucous transcriptional program to increase saliva secretion (Figure 7H).

FGF10-FGFR2b signaling is required for epithelial homeostasis, self-renewal and regeneration in basal cell progenitors in several tissues such as airways, prostate, and mammary glands^30–33^. In SGs, the current model is that basal cells are lineage restricted progenitors from the onset of differentiation, contributing to both duct homeostasis and duct regeneration^34–36^. Irradiation injury further induces plasticity in these progenitors, which leads to limited long-term regeneration of acinar cells^34^. Still, the signaling factors required to drive progenitor function in SG basal cells are not well-characterized. We recently reported that salivary ionocytes in the ducts of postnatal SG express FGF10, one of the major ligands for FGFR2b, and we predicted a potential interaction with FGFRs in adult basal cells^28^. Here, we show *Fgfr2b* deletion in basal cells leads to an abnormal duct phenotype in adult glands. Stage-specific deletion of these receptors in adult basal ducts reduced their lineage contribution, highlighting a critical role for FGF signaling in postnatal duct progenitor function similar to other stratified epithelia. Thus, we propose that ionocyte-basal cell crosstalk via FGF10-FGFR2b as a potential driver of postnatal duct homeostasis. Whether FGFR signaling is required for injury induced plasticity in SG basal cells remains to be determined.

A major challenge for regeneration of acinar cells is the identification of specific signals inducing the functional secretory cell type. Thus, several developmental studies have focused on signaling pathways and transcription factors critical for progression and timing of endbud development and specification into acinar cells. In SGs, formation of the initial bud with Sox9+ and Sox10+ distal cells is driven by FGF10-FGFR2b signaling^21,37^. By E14, combined FGFR2b and KIT signaling specifically expands the Sox10+ distal endbud progenitors and amplifies FGFR2b-dependent transcription^38^. Recently, NRG1-ERBB3 neuronal-epithelial crosstalk was implicated in acinar specification during gland development, driving onset expression of canonical acinar markers such as A*qp5* and *Bpifa2*^39^. In addition, other factors are likely involved in acinar regeneration post-irradiation damage, which was increased by neuronal stimulation that induced acinar cell proliferation^40^. However, it was also recently shown that irradiation of human SGs also leads to MEC-specific upregulation of neurotrophin receptors, which potentially hinder acinar regeneration^41^. Any regenerative therapy will need to take into consideration multiple factors that directly drive acinar specification and differentiation, as well as MEC-specific factors that influence acinar regeneration.

Here, we show the “dose-dependent” requirement of FGFR2b signaling via MAPK pathway in acinar cells drives the secretory differentiation of seromucous cells in the SMG. We genetically deleted FGFRs in cells expressing *Aqp5*, implying they were committed towards an acinar fate. Accordingly, acinar cell fate was not prevented as canonical acinar markers were still present after both FGFR1b and FGFR2b deletion, although acinar morphology and secretory protein production were severely affected. It is likely that a combination of niche factors such as EGFR and FGFR ligands may be required to initiate acinar cell fate and then drive seromucous and serous differentiation, respectively.

During embryonic development, mesenchymal cells provide the FGF ligands FGF10 and FGF7 required for epithelial development^42^. Here, we identified that MECs are one of the major sources of *Fgf7* in postnatal SGs. Considering that MECs directly contact acinar cells within the basement membrane, identifies them as critical niche cells that drive the seromucous and serous acinar secretory transcriptional profile, as well as providing contractile force to expel saliva from the acini. Further, we show expression of FGFR2b and FGF7 in adult acinar and MECs respectively, which may suggest continued acinar-MEC crosstalk via FGF7-FGFR2b as important for seromucous differentiation and function, which may stimulate secretion in adult glands. This is supported by the observation that there was increased saliva flow, evidenced by drooling mice with wet necks and chests, when FGF7 was overexpressed with the Krt14 promoter in basal cells and MECs in vivo^43^. Further, Palifermin which is a truncated version of keratinocyte growth factor 1 (KGF1 is FGF7) and an FDA-approved drug used to treat mucositis in patients receiving chemo-and/or radiation-therapy^44^, caused an increase in acinar area in histology sections and increased acinar proliferation, when injected into mouse SGs before irradiation^45^. Whether such treatment promotes seromucous and serous acinar cell types specifically remains to be determined.

Here, we have established cell-specific roles for FGFR signaling during epithelial development, duct homeostasis and seromucous and serous acinar differentiation. It is not clear whether disruption of FGFR2b signaling is directly involved in acinar pathogenesis in a setting of gland dysfunction post irradiation. However, gene therapy with retroductal infusion of an adenovirus expressing FGF7 restores salivary flow in irradiation-induced salivary hypofunction^46^. Similarly, intraglandular injections of FGF7 has protective effects of murine SGs in vivo, as well as human SG cells in vitro, following irradiation- or radioiodine-induced hypofunction^45,47,48^. The proposed mechanism in these reports include protecting acinar cells from apoptosis and stimulating the basal duct progenitor pool to differentiate into secretory acinar cells. The protective effects of FGF7 were proposed to include maintaining acinar, MEC and endothelial markers as well as saliva flow^47,48^. Taken together, these reports suggest an important role for FGF7-FGFR2b signaling in several cell types after irradiation damage and highlights potential for increasing acinar differentiation during tissue regenerative strategies.

Our findings provide genetic in vivo evidence that FGFR2b signaling will be required for stem cell-based or organoid-based regenerative therapies that will require seromucous acinar and serous differentiation as part of fully functional SG regeneration. It is likely that both or sequential EGFR and FGFR inputs will be required to drive acinar specification and subsequent seromucous acinar cell differentiation. Thus, we propose an central role for FGF7-FGFR2b signaling allowing for differentiation and maturation of specific acinar cell types, acinus morphology, as well as duct progenitor function.

### Limitations of the study

Whether there are differential roles of FGFR1b and FGFR2b in basal duct cells remains to be determined. Although implied, acinar cell secretory volume was not directly measured in mice since *ACID-Cre+;Fgfr2*^*fl/fl*^ animals did not survive more than three days after birth. Our data highlight that mucous acinar differentiation in the SLG was not FGFR-dependent, and the signaling pathways that drive mucous secretory cell differentiation remain to be determined. The effect of FGFR signaling on MEC development and function was not directly addressed in the models used here, although recent evidence shows that MEC differentiation is driven by neurotrophin signaling. Lastly, this study does not address the FGFR-dependence of cellular plasticity following injury.

## Materials and Methods

### Lead Contact

Further information and requests for resources should be directed to Marit H. Aure (marit.aure@nih.gov).

### Materials availability

No specific materials were generated in this work, see Supplemental Table 1 for resources.

### Data and Code availability

Data from scRNAseq used in this study are from previously published datasets and are publicly available (see Key resource table). Scripts generated for this study are available upon request.

## Experimental Model and Subject Details

### Mouse Strains

All mouse strains used have been previously described and included *Crect*, *Krt14Cre*, *Krt5Cre*, *Krt5rtTA;tet-Cre*, *ACID*, *Fgfr1fl/fl*, and *Fgfr2fl/fl* alleles. Reporter strains mTmG (*Gt(ROSA)26Sor*^*tm4(ACTB-tdTomato,-EGFP)Luo*^/J, The Jackson Laboratory) and tdTomato (B6.Cg-*Gt(ROSA)26Sor*^*tm9(CAG-tdTomato)Hze*^/J, The Jackson Laboratory) in addition to timed pregnant ICR (CD-1^®^) females (Envigo) were also used. Mice carrying *Fgfr1*^*fl/fl*^ and *Fgfr2*^*fl/fl*^ were mated and subsequently maintained as a double floxed strain. To generate the *Fgfr1* ^*fl/fl*^ *;Fgfr2*^*fl/fl*^ embryos used in the study, timed mating was set up, and the morning of the day a plug was detected was considered day 0. Genotyping was performed using standard protocols (See Supplemental Table 1 for specific primers and mouse strains). All experiments were approved by the NIH Animal Care and Use Committee.

### Doxycycline treatment

Adult mice (6–8 weeks old, males and females) were fed Doxycycline diet (5001C w/6000ppm, Animal Specialties and Provision, PA, USA) ad libitum for 4 days (day 0), before changing the food back to standard diet. Tissues were harvested for analysis at indicated timepoints. For induction during embryonic development, females were fed doxycycline during pregnancy.

### Organ Culture Explants

SMGs were dissected from ICR embryonic day 15 (E15) embryos and placed on Whatman Nuclepore Track-etch filters (13 mm, 0.1 μm pore size; VWR, Buffalo Grove, IL) with 200μL fresh DMEM/F12 media (from Thermo Fischer Scientific) containing 1% Penicillin-Streptomycin, Transferrin (150μg/ml, from Thermo Fischer Scientific) and Vitamin C (50μg/ml, from Sigma Aldrich). Two to three glands were placed per filter and allowed to set for 2 hrs before addition of inhibitors targeting FGFR signaling and its downstream effectors. Inhibitor stocks of SU5402 (Millipore Sigma), UO126 (Millipore Sigma), U73122 (Millipore Sigma), and Ly249002 (Biotechne) were resuspended in DMSO (Sigma Aldrich) as per vendors instructions. Full 5, 20, 20, and 25μM inhibitor dosages respectively were made after forming an intermediate solution diluted with DMSO. Glands from at least 3 independent litters were included for each treatment group (n=3). Experimental groups were run along with equivalent vehicle (DMSO) as control as well as a group of untreated glands. SMGs were cultured at 37°C in a humidified 5% CO_2_/95% air atmosphere for 24h (unless otherwise noted). RNA was isolated from homogenized glands before qPCR gene expression analysis as described below.

Organ culture experiments with washout and ligand treatments were cultured under the same conditions as described above. Here, glands were treated with UO126 (20μM) or vehicle (DMSO) for 24hrs before washout with fresh media 3×5 minutes. Glands were then incubated again for 24h with either UO126, DMSO, FGF7, and FGF10 (All recombinant FGF’s from R&D Systems). Gentle but thorough mixing was done to assure adequate distribution of treatment in the media. Final dosages were 500ng for FGF7 and FGF10 (volume of 10μL).

### Real time qPCR

RNA was isolated using either RNAqueous-4PCR total RNA isolation kit or RNAqueous-PCR micro kit with DNase treatment (Both from ThermoFisher). cDNA was made using the iScript cDNA Synthesis Kit (Bio-Rad) and 1 ng was amplified with 40 cycles of 95°C for 10 s and 62°C for 30 s. Gene expression was normalized to the house-keeping gene, *Rps29*. Amplification of a single product was confirmed by melt curve analysis and all reactions were run in duplicate.

### Immunohistochemistry

For frozen sections, tissues or whole embryos were fixed in 2% paraformaldehyde (Electron Microscopy Sciences, #15700) overnight (ON), then stored in PBS. Tissues were dehydrated with increasing sucrose concentrations, 15%, 30%, and then 1:1 30% sucrose: OCT^™^, until equilibrated, then embedded in Tissue-Tek^®^ OCT^™^ compound (Sakura) at 0°C. Frozen sections of 10 μm or 50 μm were cut and placed on Superfrost Plus glass slides (Thermo Fisher Scientific). After blocking with 10% Normal Donkey Serum for 1 hr at room temperature (RT), sections were incubated with primary antibody ON at 4°C followed by appropriate secondary antibody incubation for 1 hr at RT. Cover slips were mounted using Fluoro-gel II, with DAPI (#17958–50, Electron Microscopy Sciences) and set overnight at room temperature. Sections were washed in PBS between each step.

For paraffin sections, tissues were fixed in 4% paraformaldehyde (Electron Microscopy Sciences, #15700) ON and transferred to 70% ethanol until day of embedding. Tissues were embedded in paraffine, and sections cut following standard procedure (Histoserv Inc., Germantown, MD). For staining, sections were deparaffinized in Xylene substitute (Sigma Aldrich, A5597–1GAL) followed by hydration through a graded series of ethanol (100–95-70%) to H_2_O. The slides underwent heat-induced antigen retrieval using a Tris-EDTA pH 9.00 buffer (TrisBase T1378, EDTA E9884 from Millipore Sigma) using a pressure cooker. Slides were allowed to cool down for 1 hr at RT followed by 1 hr incubation in either normal donkey serum (Jackson Immunoresearch Laboratories) or M.O.M block (Mouse on Mouse Immunodetection Kit, Vector Laboratories, BMK-2202). Primary antibodies were incubated ON at 4°C followed by 1hr incubation in secondary antibody. Sequential staining was done if several primary antibodies were used. Nuclear staining with Hoechst (Thermo Fisher Scientific, H3570) was done and coverslips were mounted using Thermo Scientific^™^ Shandon^™^ Immu-Mount^™^ (#9990402). Slides were washed in PBS between each step.

Co-staining done with same species antibodies were performed using a multiplex method. Paraffin sections were de-paraffinized as described above before antigen retrieval in a microwave (2:30 mins at 700W power followed by 5 minutes at 300W power) in Tris-EDTA pH 9.00 buffer before 1 hr cool down. Slides were incubated 10 minutes in Bloxall^®^ Endogenous Blocking Solution (Vector Laboratories, SP-6000) before primary antibody incubation at 4°C ON. Slides were rinsed in PBS and incubated 30 minutes at RT in the appropriate VisUCyte HRP Polymer Antibody (R&D systems). Sections were rinsed in PBS and treated with pre-incubation buffer (0.1M Boric Acid, 2M Salt Chloride, 0.2 mg/mL 4-Iodophenylboronic acid) for 10 minutes before 10 minutes with the appropriate Tyramide reagent (diluted in buffer: 0.1M Boric Acid, 2M Salt Chloride, 0.2 mg/ml 4-Iodophenylboronic acid and 0.003% H2O2). Antigen retrieval step and staining steps were then repeated until all antibodies had been stained. Nuclear staining and cover slips were mounted as described above.

Hematoxylin and Eosin (H&E) staining of paraffine sections were performed by Histoserv Inc (Germantown, MD). Slides were scanned with a S60 NanoZoomer Digital (Hamamatsu) and images were exported using the NDP.view 2 software (Hamamatsu).

### In situ Hybridization

Using RNAse free solutions, SMGs from P1 and adult ICR mice were isolated, washed in PBS and fixed in 4% PFA before paraffine embedding. Sections were sent to Advanced Cell Diagnostics (ACD) for in situ hybridization for *Fgfr1*, *Fgfr2*, *Bhlha15*, *Krt5, Cnn1, Pdgfra, and Fgf7*. Specific probe sequences are proprietary and generated with RNAscope^®^ technology by ACD.

### Computational analysis

Using previously annotated mouse SMG scRNAseq data, epithelial populations were computationally separated SEURAT’s subset function. Epithelial subsets from E12 were re-normalized and scaled to generate new SEURAT objects. E16 and P1 acinar subsets were integrated through standard pipeline to generate a new SEURAT object with mouse acinar cells. All statistics for the computational analyses to determine significant markers were performed using the default pipeline statistical test in SEURAT. This analysis is based on non-parameteric Wilcoxon rank sum test and adjusted p-values of <0.05 were chosen as a measure of significance. Human MSG datasets (GSE180544 and https://www.covid19cellatlas.org/) were integrated and previous annotation from the two datasets were used to identify cell populations. Human SMG and PG datasets were imported (GSE201333) and the previous annotations were used to identify populations.

### Quantification of histological images

Images were taken from random areas of H&E-stained *Krt5Cre; Fgfr1*^*fl/fl*^*; Fgfr2*^*fl/fl*^ slides as described above. Duct/total area ratio was calculated by measuring the area of duct and total area using FJII [8]. For quantification of fluorescent images, imaging was performed using a 40x objective on a Nikon A1R+ MP microscope using resonant scanning method. From *Krt5rtTA; tetCre;Fgfr1*^*fl*^*;Fgfr2*^*fl*^*;mTmG* sections 10 random areas were selected (5μm thick confocal stacks with 0.5μm steps) from at least 3 different animals (n=3). The GFP and Hoechst expression intensity was measured by calculating the integrated density value of the samples histogram resulting from the maximum intensity projection. Quantification of the 10 points were averaged and GFP was normalized to Hoechst staining. Results were then averaged by biological replicates. For quantification of Ki67, Cleaved Caspase3, SMGC and LPO, 2–3 confocal stacks (5μm stacks, 0.5 μm steps) from 3 glands (n=3) were processed using the denoise function of the Confocal NIS-Elements Package prior to quantification. Threshold with stack histogram was set manually to reduce background, and integrated density was measured on maximum projections and normalized to Hoechst (using FIJI). All quantification measurements of confocal imaging were performed blinded.

### Statistical analysis

To compare more than two experimental groups, we performed one-way ANOVA with post hoc Dunnett’s or Tukey test (as indicated) and for comparison of two data sets, the Student’s t-test with two-tailed tests and unequal variance was used to calculate p-values. Graphs show mean ± SEM for each group from three or more replicates.

## Highlights

- Deletion of FGFR2b in basal duct progenitors reduces lineage contribution during homeostasis.
- Acinar-specific deletion of FGFR2b inhibits SMG seromucous and SLG serous acinar differentiation.
- FGFR2b signaling regulates seromucous acinar differentiation via the MAPK pathway.
- MECs are an endogenous source of FGF7 and exogenous FGF7 activates the seromucous transcriptional program.
